# Embedding sports and exercise medicine into the medical curriculum; a call for inclusion

**DOI:** 10.1186/s12909-018-1422-9

**Published:** 2018-12-13

**Authors:** Tej Pandya, Katherine Marino

**Affiliations:** 1 Faculty of Biology, Medicine and Health, University of Manchester, Manchester, UK; 2 University of Sheffield Medical School, University of Sheffield, Sheffield, UK

**Keywords:** Sports medicine, Exercise medicine, Medical education, Medical curriculum

## Abstract

The UK is currently facing an inactivity crises, with 1 in 5 children currently meeting physical activity guidelines (Health Survey for England, Children’s Health, 2016). To combat this growing problem there has been increased interest in promoting exercise and healthy lifestyle advise to patients as a method for improving public health. In line with this, the specialty of Sports and Exercise Medicine (SEM) has been gaining momentum and is now a recognised specialty with a higher specialist training programme. This postgraduate speciality aims to produce doctors who are experts in exercise and musculoskeletal medicine. Increasing numbers of NHS departments are employing SEM doctors to better manage musculoskeletal (MSK) issues and prescribe exercise (Morrissey et. al, Muscles Ligaments Tendons J 3:190–195, 2013). In keeping with this increased opportunity for SEM in postgraduate training, we believe that we should not forget that SEM should not be exclusive to postgraduates and there is increasing interest and need for teaching to medical students (Cullen et al, Br J Sports Med 34:244-245, 2000). This article provides an overview to students and clinicians into the current state of undergraduate SEM education in the UK, and highlights the importance of incorporating SEM into the medical curricula.

## Main text

Several studies have shown that medical students want more exposure to the speciality and therefore we believe that there is currently not enough teaching of SEM in medical schools [1–3]. Indeed, only 52% of a sample of UK final-year medical students state they feel comfortable delivering physical activity advice [4]. Inactivity is a significant risk factor for disability and death, and to combat this as a society current public health strategy involves promoting physical activity as a form of medicine [5, 6]. Furthermore, it remains reasonable to hypothesize that as we successfully encourage more patients to get active, it is possible more will also present with activity-related MSK issues. Consequently, clinicians skilled in prescribing exercise medicine and dealing with MSK issues will be ideal to deal with this workload. The lack of exposure to the SEM speciality results in a lack of awareness of what is involved in SEM, including training schemes and its place in health service provision [7]. Developing the interest of undergraduates and junior doctors is necessary for the sustainability and future of the SEM specialty.

We believe medical schools in the UK need to better educate their students on how physical activity is essential for their patient’s health and have the necessary skills and knowledge to promoting it to patients. Several universities across the world have successfully incorporated SEM into their curriculum, with a focus on exercise medicine and how to successfully prescribe this in patients, and we need to learn from these to develop best practice [8–10]. Some medical schools in the UK have been successfully starting to integrate SEM into their medical curriculum [11]. However, currently the majority have it incorporated as optional modules rather than compulsory teaching for all medical students.

The growing epidemic of MSK related presentations needs to be addressed, and in response to this the NHS have introduced pilots of first contact practitioners, which are usually physiotherapists, to manage these conditions, without them having to be referred by the GP [12]. Initiatives like these may help reduce the burden of MSK issues from GPs, however it is still essential that GPs have the skills needed to deal with MSK issues and work with physiotherapists in initiatives like this to provide high quality MSK services.

To try and improve the current situation Public Health England and Sport England have organized a project to visit medical schools to develop frameworks for embedding physical activity into curriculums, and they acknowledge that medical students can help encourage medical schools to do this [13]. For students that are interested in SEM there are limited options provided by medical schools to learn more about the speciality. Many students must take a year out of medical school to do an intercalated degree in an SEM related subject.

Student-led activities in SEM however, are thriving. The British Association of Sport and Exercise Medicine (BASEM) student memberships are at record number and undergraduate SEM societies have been established at 79% (*n* = 26) of UK medical schools [14, 15]. This growing network of undergraduate societies is overseen by the undergraduate section of BASEM: the Undergraduate Sports and Exercise Medicine Society (USEMS). Ten USEMS conferences have been held to date, cumulatively attracting over a thousand students. In addition, there are now several platforms available for students to simultaneously engage with the specialty and educate others, such as through British Journal of Sports Medicine (BJSM) blogs and the USEMS eMagazine. To engage students with research an annual SEM poster competition open to all UK undergraduates was setup in 2011. The highest number of entries has come from institutions that offer intercalated degrees in sports medicine and have large undergraduate sports medicine societies, and the subsequent publication of this work in peer reviewed journals highlight the high calibre of work that can be achieved by undergraduates given the correct mentorship and opportunity [16].

With relation to the ‘sport medicine’ side of SEM, many have the incorrect perception that SEM is only about managing elite athletes. In reality, SEM doctors are highly skilled in managing sport-related pathologies in the general population as well as elite athletes. MSK issues currently account for one in seven consultations in General Practice (GP) and evidence shows that GPs do not feel that they have enough training to deal with MSK related pathologies [17]. The most common barrier to incorporating SEM into medical school curricula is a lack of space and time and finding space will undoubtedly be difficult [4]. However, we believe it is of upmost importance we prepare future clinicians will the skills necessary to improve the healthcare of patients through physical activity advice, and have the skills to deal with an increase in MSK injuries that this might result in. Therefore, we put forward the argument that medical schools have a duty to incorporate more SEM into the undergraduate medical curriculum.

## Conclusions

Undergraduate SEM in the UK is growing in interest and commitment and there any plenty of extra-curricular opportunities for students to get involved in the specialty. It is time now, to embed the principles of MSK and exercise medicine into the undergraduate curriculum. It is more important than ever that the next generation of doctors are more aware of the role that SEM can play in improving quality of life and preventing and treating disease. With a flourishing student base, there is potential for students to influence change and incorporate SEM into good medical practice. This has got to be good news for patients too!

## Abbreviations

BASEM: The British Association of Sport and Exercise Medicine
MSK: Musculoskeletal
SEM: Sport and Exercise Medicine
USEMS: Undergraduate Sports and Exercise Medicine Society
